# Scale free is not rare in international trade networks

**DOI:** 10.1038/s41598-021-92764-1

**Published:** 2021-06-25

**Authors:** Linqing Liu, Mengyun Shen, Chang Tan

**Affiliations:** 1 Economics and Management School, Wuhan University, Wuhan, 430072 China; 2 College of Economics and Management, Southwest University, Chongqing, 400715 China

**Keywords:** Complex networks, Statistical physics

## Abstract

Failing to consider the strong correlations between weights and topological properties in capacity-weighted networks renders test results on the scale-free property unreliable. According to the preferential attachment mechanism, existing high-degree nodes normally attract new nodes. However, in capacity-weighted networks, the weights of existing edges increase as the network grows. We propose an optimized simplification method and apply it to international trade networks. Our study covers more than 1200 product categories annually from 1995 to 2018. We find that, on average, 38%, 38% and 69% of product networks in export, import and total trade are scale-free. Furthermore, the scale-free characteristics differ depending on the technology. Counter to expectations, the exports of high-technology products are distributed worldwide rather than concentrated in a few developed countries. Our research extends the scale-free exploration of capacity-weighted networks and demonstrates that choosing appropriate filtering methods can clarify the properties of complex networks.

## Introduction

Scale-free networks were first introduced in 1999 by Barabási and Albert^1^ after testing of many real networks in different fields by verifying that their degree distributions follow power law. Scale-free structure has since been widely observed in the real world^2–4^. The power-law shape of degree distribution is an important feature of scale-free networks that imparts many unique characteristics compared with random networks, such as robustness to random failures and fragility to targeted attacks. However, nearly 20 years later, Broido and Clauset (B&C) claimed that scale-free networks are rare based on an analysis of nearly 1,000 real networks^5^. Barabási refuted B&C’s conceptual and technical critique by posting *“Love is all you need”* on his homepage^6^. Despite the dispute provoked by B&C’s article, investigations of the existence of scale-free networks^7–10^ and explorations of methods to test power-law^11^ distribution continue. For example, recent research by Serafino et al. supports the claim that complex networks are inherently scale-free through finite-size scaling analysis^12^. Although Serafino et al. reached a different conclusion than B&C, their power-law fitting results for social networks were similar. In Serafino et al.’s study, 69% of social networks were not scale-free^12^, while 50% were scale-free in B&C’s research^5^. A social network is a typical weighted network where the correlations between weights and topology are very important for understanding the characterization of real network properties^13^. Ignoring weights or applying inappropriate methods to weighted networks can lead to biased results when exploring the scale-free property.

When the networks were multiplex, bipartite and multigraph, B&C ignored the weight; when the networks did not have multiplex, bipartite and multigraph properties, they used the principle of the largest-weighted edges (LWE) to simplify weighted networks. According to our replication of LWE applied to international trade data, we argue that LWE limits the exploration of the scale-free property (more details in Supplementary Note 1):

1. LWE cannot produce networks with desired mean degrees;
2. The simplified networks are still dense and have mean degrees much higher than desired;
3. The number of nodes (countries) is drastically reduced after simplification by LWE.

We suppose that the abovementioned limitations are the reason that the scale-free structure cannot be observed. For weighted networks, there is considerable heterogeneity in the capacity and intensity of edges^13^, as edge weights can be assigned differently. In a capacity-weighted network, the definition of edge weights is a straightforward and objective measure of the capacity or flow of edges, whereas in an intensity-weighted network, edge weights represent the strength of the connection between two nodes determined through complicated calculations^13–15^. In the case of a capacity-weighted network, the correlation between weight and topological properties is particularly strong (more details in Supplementary Note 2):

1. The distribution of weights and the distribution of strengths follow an approximate power law with a heavy tail;
2. The strength of nodes, simply measured as the total flow through each node, grows faster than their degree. Thus, the higher the degree of a node, the higher the capacity of the connected edges;
3. High-degree nodes have a progressive tendency to form interconnected groups with high-flow connections. In other words, as high flow is mostly associated with hubs, high-degree nodes are more likely to form cliques with nodes of equal or higher degree according to the so-called *rich-club phenomenon*^16^.

Based on the abovementioned characteristics of capacity-weighted networks, we propose an optimized simplification method by combining Zhou et al.’s top network method^17^ with previous research findings. Our research aims to improve methods for the simplification process of capacity-weighted networks. We find that LWE fails to observe scale-free features in capacity-weighted networks for the following reasons:

1. Because of the lack of a characteristic scale due to the heavy-tailed distribution of weights, any method based on a threshold simply overlooks the information present above or below the arbitrary cutoff scale^18^;
2. The strong correlations between weights and topological properties not only causes high-strength nodes to attract high-degree nodes with high-flow edges but also makes effective preferential attachment possible with weight-driven growth^19^.

In the “Results”, by using our simplification method and classification of scale-free, we test the power-law behavior in the degree distribution in international trade networks (ITNs). First, we assume that each type of network should be analyzed specifically and that the analysis should be concerned with the network property itself; thus, we choose one type of network for testing. Second, an ITN, as a typical capacity-weighted network, has edge weights directly measured by trade value. This feature is appropriate for verification. Third, previous studies have mostly taken the trade volume of all products as a connection between two countries. By accounting for product heterogeneity, we extend the analysis to the product level.

## Results

We generated more than 1200 product trade networks each in imports, exports and total trade for the study period 1995 to 2018. To avoid the complications derived from trade flow imbalances^13^ and to take into consideration the heterogeneity of imports and exports, we build an undirected import network (IMN), an undirected export network (EXN) and an undirected total trade network (TTN) based on each product trade dataset (see “Data” for details). A resulting product network has ${\text{N}}$ nodes denoting countries and ${\text{E}}$ edges accounting for the presence of trade relations. In case of IMN, the edge weights $w_{{ij}}$ of an edge linking country ${\text{i}}$ and country ${\text{j}}$ are straightforwardly defined as the trade value of exports from ${\text{i}}$ to ${\text{j}}$, and the adjacency list is $a_{{ij}}$. In contrast, $a_{{ji}}$ is the adjacency list for EXN, and the edge weights $w_{{ji}}$ represent the trade value of exports from country ${\text{j}}$ to country ${\text{i}}$. For TTN, we sum the trade value of exports and imports between each pair of countries. If a trade relation exists, the trade value sum is taken as the edge weights in the adjacency list.

In addition, we build entire trade networks by summing the trade value of all products. Similar to the product network generating process, we add up the trade value of all products’ exports from country ${\text{i}}$ to country ${\text{j}}$ and build IMN. In contrast, the edge weights of EXN are defined as the trade value of all products’ exports from country ${\text{j}}$ to country ${\text{i}}$. For TTN, we sum the trade value of all products’ exports and imports.

### Simplification by Top N filtering

The next step is to simplify product trade networks to obtain a simple graph, and then, the scale-free hypothesis can be defined clearly with the degree distribution^5^. B&C used the LWE simplification method to transform a network by using three thresholds^20^. However, the simple thresholding algorithm has two serious disadvantages when simplifying ITN:

1. Since most of the high-degree nodes (countries) also have high flows (trade value), the cutoff obtained by LWE in capacity-weighted networks drastically removes all information below the cutoff scale^18^. For ITN, this means that newly established trade relations, which mostly have low trade value, will not be included after simplification.
2. In ITN, there is a rich-club phenomenon^21^, and edge weights are distributed towards a power law^22^ (more details in Supplementary Note 2). As a result, nodes (countries) with small weights (trade value) are systematically neglected when using a simple thresholding algorithm^18^. This implies that low-degree countries cannot be retained in simplified networks.

To improve the simplification method for capacity-weighted networks, we introduce and extend Zhou et al.’s top network method^17^. This method, Top N Filtering, ranks nodes (countries) by the magnitude of their edge weights (trade value) for each node (country), and we retain only the top N ranked edges (trade relationships), namely, the N largest neighbors (trade partner) of a node (country). B&C evaluated whether this network was scale-free after simplification by analyzing what percentage of simple graphs can meet the requirements^5,20^. However, Barabási argued that only one of these graphs matters: *“the one that captures the purpose or the function of the original system”*^6^. Thus, we argue that the mean degree $k = 2$ is reliable for observing scale-free structure in the simplified networks; this value was not only proposed by B&C as the lowest threshold in a simple graph^11^ but also lies in the reasonable range of mean degrees recommended by Barabási to observe scale-free networks^23^. Thus, Top N Filtering has three advantages over a simple thresholding algorithm:

1. First, it accounts for the correlation between the weights and topology of the network (see Supplementary Note 2 for details). Regarding these two features of capacity-weighted networks mentioned in “Introduction”, the simple thresholding algorithm neglects the low trade flows and the low-degree countries, whereas Top N Filtering guarantees that all nodes (countries) are included and equally represented in the constructed network^17^.
2. Second, the number of trade relations is controlled, and the simplified networks can have the desired mean degree $k = 2$ when let ${\text{N}} = 1$ (see Table 2–4 in Supplementary Note 3). Thus, the obtained networks after simplification are sufficiently sparse to observe their scale-free nature.
3. Third, the simplified networks based on the top relations reflect countries’ preferences in choosing trade partners^17^. Considering the limited number of countries in ITN, the preferential attachment mechanism in ITN reflects not only the ability to attract new countries to build trade relations with high-degree countries but also increasing weights on existing trade relations.

After simplification of all product trade networks by using Top N Filtering, we applied standard statistical methods to identify the best-fitting power law in the degree distribution’s upper tail and compare it to three alternative distributions fitted to the same part of the upper tail using a likelihood-ratio test (see “Methods” for details). These three models contain log-normal, exponential and Poisson distributions. In total, we obtain 30,185 fitting and comparison results for the EXN, 30,311 results for the IMN and 30,289 results for the TTN, which will be evaluated according to our definition of scale-free for a capacity-weighted network.

### Definition of scale-free

In response to the above modification of the network simplification process, we adjusted the definition of scale-free for capacity-weighted networks. In line with B&C^5^, Voitalov et al.^7^ and Serafino et al.^12^, we also define a classification for the degree distribution of capacity-weighted networks on the basis of tests. For example, in addition to considering the fitting results of the power-law model, B&C also included the comparative results between the power-law and three alternative distributions to classify scale-free networks into 6 levels (see Supplementary Note 4). Based on previous studies, we define our first type of scale-free network as direct evidence. The power law is itself a fitted model of degree distributions and is defined as follows.

- Standard:
- A power-law distribution cannot be rejected (p >  = 0.1);
- The power-law region contains at least 50 nodes (n_tail_ >  = 50); and
- The estimated scaling parameter meets the requirement:$2 < \hat{\alpha } < 3$.

In addition to direct evidence, the second type also includes indirect evidence that the observed degree distribution is not required to be plausibly scale-free, but no alternatives are favored over the power law. We define it as:

- Advanced: Based on Standard, no alternative distribution is favored over the power law.

**Figure 1:**
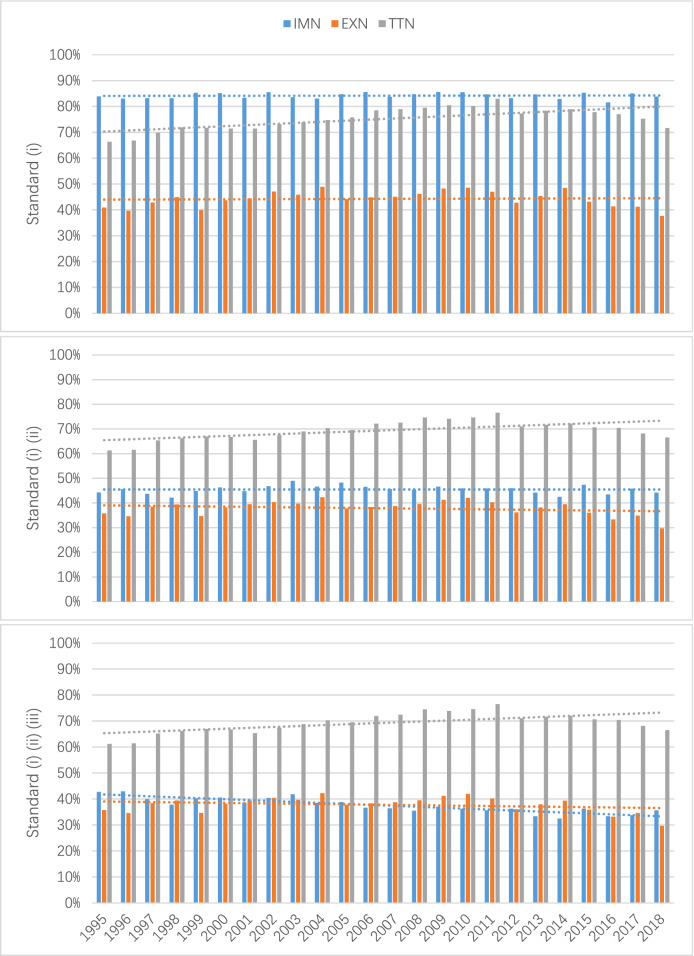
Proportion of products that meet the Standard level of scale-free in IMN (blue), EXN (orange) and TTN (gray).

Figure 1 shows the annual proportion of product networks in the EXN, IMN and TTN that gradually meet the standard level of scale-free. Obviously, there are many more products in the TTN than the IMN and EXN that can meet all three standard requirements in each year. The development of the EXN, IMN and TTN from 1995 to 2018 was relatively slow. In the EXN and TTN, we observe small differences in quantity across the three requirements. However, over 80% of products in the IMN can mostly satisfy the first requirement, but nearly half of them are eliminated after considering the second standard requirement (whether more than 50 nodes remain in the power-law region). In essence, we observe a scale-free structure in parts of product trade networks under the standard criteria.

For the entire trade network, we find that the studied periods 2004–2008 and 2010–2016 for IMN meet the standard requirement for scale free, while no studied year for EXN and TTN is scale-free (see more details in Supplementary Note 5). Further analysis on scale-free IMN in the abovementioned study years shows that the power-law model is favored over exponential and Poisson distributions, but the test outcome is inconclusive between power-law and log-normal distributions.

### Alternative distributions

Across the product trade networks, our test finds only modest support for the power-law distribution over the three alternative distributions (see Tables 6–8 in Supplementary Note 6). For almost all product trade networks, their power-law distributions are not favored over log-normal distributions (M_PL_ in Tables 6–8 in Supplementary Note 6). Furthermore, the test outcome is inconclusive between power-law and log-normal distributions for approximately half of product networks in the EXN and IMN (M_inconclusive_ in Tables 6–7 in Supplementary Note 6). In the TTN, M_inconclusive_ increased from 72 to 81% over the studied period, which implies limited differences between the power-law and log-normal distributions. In contrast, power-law distributions are generally better than exponential distributions and Poisson distributions, regardless of whether we consider the EXN, IMN or TTN (M_EX_ and M_PO_ in Tables 6–8 in Supplementary Note 6). In essence, regardless of whether the power-law model is a statistically good model of the degree distribution, the non-power-law alternatives are hardly better.

Based on the evaluation using the standard criteria, we analyzed products in the EXN, IMN and TTN by considering alternative distributions’ comparative results. The outcome indicates that all standard products also meet the requirements of the second type (advanced criteria).

### Trends

**Figure 2:**
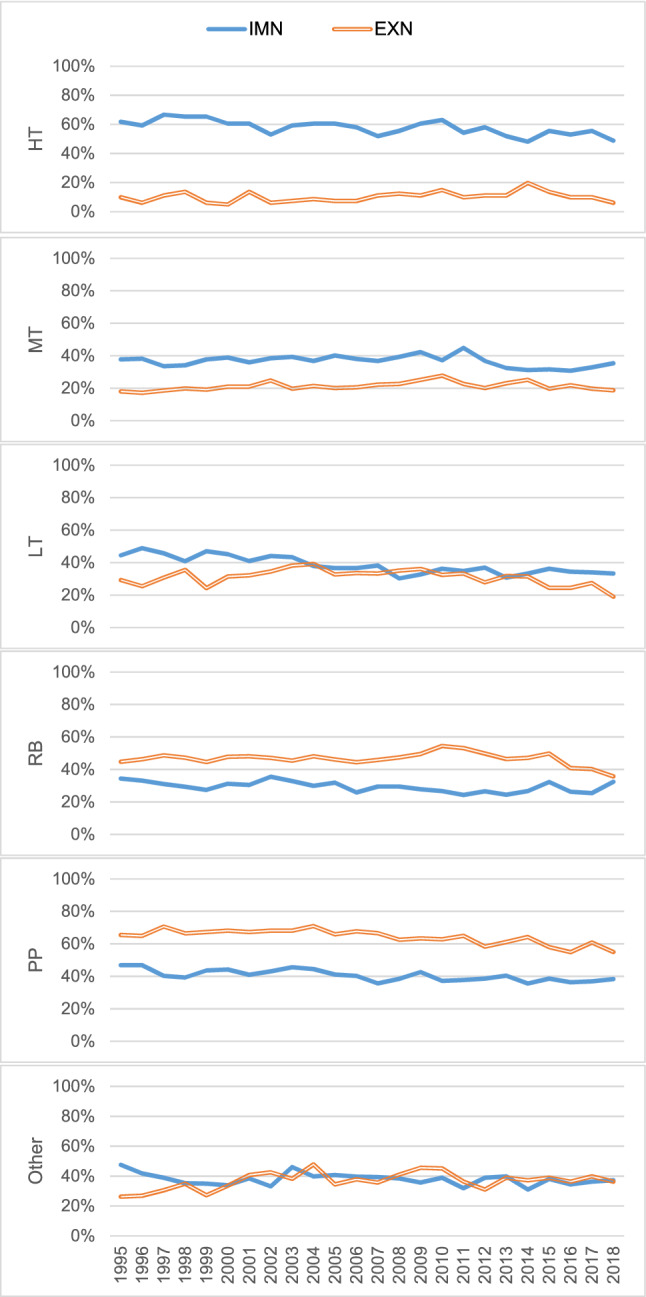
Development of the proportion of scale-free products in IMN (blue) and EXN (orange) from 1995 to 2018.

A technological classification of products created by Lall is widely used in the study of international trade. It includes the following categories^24^: primary products (PP), resource-based products (RB), low-technology products (LT), middle-technology products (MT), high-technology products (HT) and other transactions. We show in Fig. 2 the development of the proportion of scale-free product networks within their technological classifications during the studied period.

- Much more HT are scale-free in IMN than in EXN. In other words, only a few HT networks have hubs in EXN, but many have hubs in IMN. In addition, there is a big gap between IMN and EXN. The proportion of scale-free HT networks decreases slightly from 1995 to 2018 in IMN but remains relatively steady in EXN.
- More MT are scale-free in IMN than in EXN. The proportion of scale-free product networks is relatively steady in the studied period. Compared to HT, the gap between IMN and EXN is smaller for MT.
- The proportion of scale-free LT networks ranges from 20 to 40% in EXN and from 30 to 50% in IMN. This proportion decreases slowly in IMN but is quite stable over the years in EXN.
  More RB are scale-free in EXN than in IMN. Compared with HT and MT, fewer RB are scale-free in IMN but more RB are scale-free in EXN.
- Fewer PP are scale-free in IMN than in EXN. Compared with scale-free LT networks, the development of the proportion of scale-free PP networks shows an opposite trend. This proportion decreases slowly in EXN but is quite stable in IMN.

Our results demonstrate that the heterogeneity of products can lead to different topological properties of ITN. The scale-free structures of all product categories are distinct from each other. Figure 2 reveals that as the technology level decreases (from HT to RB), the proportion of scale-free products in IMN gradually decreases, while the proportion of scale-free products in EXN increases. An interesting finding is that far fewer HT products in the EXN have a scale-free structure than in the IMN. This implies that there are only a few HT product networks that have hubs in the EXN. In the ITN, hubs represent high-degree countries with which many other countries prefer to create trade relationships and enhance cooperation. Normally, HT products need more advanced technologies, and only developed countries possess the ability to produce and export them. Thus, the exports of HT products should be concentrated in developed countries, and we should find some hubs in the EXN. However, our results present the opposite: less than 20% of HT product networks have hubs in the EXN. Such a counterintuitive phenomenon demonstrates that explorations of network properties should be concerned with the characteristics of the real network itself.

We suppose that due to investment in developing countries and the production there being deeply embedded in the global value chain, an increasing number of countries are able to produce and export HT products. In contrast, many HT product networks have hubs in the IMN. This means that the imports of HT products are concentrated in only a few countries. The proportion of scale-free product networks within the HT classification decreased gradually from 1995 to 2018. We suppose that intermediate products classified as HT are widely required, and thus some countries with large production capacity need to import large quantities of HT products.

## Discussion and conclusion

Our research stemmed from our interest in exploring why previous studies have not found social networks, as typical capacity-weighted networks, to be scale-free. To answer this, we repeated B&C’s process. We noticed that their simplification methods are inappropriate for capacity-weighted networks due to two drawbacks. Thus, it is reasonable to doubt the result of a scale-free network while studying capacity-weighted networks by using B&C’s methods. To simplify capacity-weighted networks, we optimized the simplification method by introducing Top N Filtering. In making this improvement, we considered the special characteristics of ITNs: the edge weights (trade value) between existing high-degree nodes (countries) increase faster than edge weights (trade value) between new low-degree nodes, which reflects different preferential attachment. Preferential attachment is an important mechanism leading to a scale-free outcome in the Barabási-Albert model. If a new node is free to choose between a high-degree node and a low-degree node, it is more likely to connect to the high-degree node. This process leads to a faster increase in the degree of high-degree nodes than in the degree of low-degree nodes. However, in a capacity-weighted network, the network topology relates more to edge weight than to node degree. As Barabási argued, without taking the theory of real systems into account, it makes no sense to fit a pure power law to them^6^. We argue for his view since without consideration of the correlation between weights and topology, it is difficult to understand the characterization of real networks.

We tested Top N Filtering on ITNs, namely on an IMN, EXN and TTN. Our research reveals that Top N Filtering can solve problems that LWE cannot. Our research reveals that TN can solve problems that LWE cannot. In addition to the ITNs studied in this paper, there are many other capacity-weighted networks in the real world, such as airline networks, which grow with an increased number of flights and passengers, and road transportation networks, which grow by an increased number of trips and freight volume. Furthermore, we provided an overview of the scale-free structure of ITNs at the product level over 24 years. Through technology classification, we showed the development trends in different industries. For example, for HT products, there are fewer countries dominating the export market, but for PP, there are many more. Our ITN results show that scale-free is neither as widespread as Barabási contends nor as rare as B&C claimed. Each country has its own export advantage; thus, different product networks may perform differently. Product heterogeneity may be a reason for their different scale-free results. We suggest that the characteristics of a network itself should be considered when exploring scale-free structures. Developing a specialized method for each type of network is helpful in studying scale-free properties.

## Methods

### Data

We use BACI data on the CEPII database, which is built from data directly reported by each country to the United Nations Statistical Division (Comtrade)^25^. It provides disaggregated data on bilateral trade flows for more than 5000 products and 200 countries^25^. BACI offers yearly data at the product level identified by the 6-digit Harmonized System (HS). Each trade flow is characterized by a combination of exporter-importer-product-year with its trade value and quantity^25^. When the first 4 digits of HS-6-digit codes are the same, their corresponding product trade values can be summed and grouped into one HS-4-digit product category. We refer to the product category in our study simply as the product.

### Model

The model of the degree distribution applied by B&C to estimate the best-fitting power-law distribution is the form^5^

$$\Pr \left( k \right) = Ck^{{ - \alpha }}$$

where α is the scaling exponent, C is the normalization constant, and k is the integer value^5^. Typically, the scaling parameter lies in the range 2 < α < 3^5^. We started with at least k_min_ ≥ 1, where the upper tail begins, and then estimated the scaling exponent on the truncated data, as B&C did in their study^5^. To determine how likely it is that the data sets fit the power law, a standard goodness-of-fit test was applied and returned a standard p-value^5^. If *p* < 0.1, then we rejected the power law as a plausible model of the degree sequence, and if *p* ≥ 0.1, then we failed to reject the model^5,26^.

In addition, we compared the power-law distribution with three alternative models: log-normal, exponential and Poisson distributions. Each power-law model was compared to three alternative models estimated via maximum likelihood to the same degree using a standard Vuong normalized likelihood ratio test^5^. The results provide indirect evidence based on the test statistic ${\mathcal{R}}$ regarding whether the power-law model can be favored over other alternative models^5^. The sign of ${\mathcal{R}}$ shows a better fit of the power-law model (${\mathcal{R}} > 0$), the alternative model (${\mathcal{R}} < 0$), or neither model (${\mathcal{R}} = 0$)^5, 26^. A standard two-tailed test against the null hypothesis of ${\mathcal{R}} = 0$ gives a p-value that can tell us whether the observed sign of the logarithm of the ratio is statistically significant^5^. If *p* < 0.1, then this sign is a reliable indicator of which model is the better fit^26^. If *p* ≥ 0.1, the result is not reliable, and the test result does not favor either model over the other^26^.

## Supplementary Information

Supplementary Tables.
