# Supplementary material for: Scale free is not rare in international trade networks
Source: Sci Rep. 2021 Jun 25;11:13359. doi: 10.1038/s41598-021-92764-1 (PMC8233329; doi:10.1038/s41598-021-92764-1)
Supplement: Supplementary file 1 — Supplementary Tables. [file 41598_2021_92764_MOESM1_ESM.docx]

Supplementary Note 1: Simplification by LWE

In Broido and Clauset’s supplementary notes, they provide two paths for simplifying network data to degree sequences, as shown in Figure 1. Path A treats the data by ignoring the weighted edges for multiplex, bipartite and multigraph networks^1^. Path B illustrates that the weighted networks are transformed to simple graphs according to three mean degree thresholds. In other words, by choosing the $m=\left\{ n, \frac{1}{2}n^{\frac{5}{4}}, \frac{1}{2}n^{\frac{3}{2}} \right\}$ largest-weighted edges, the resulting graphs should have mean degree$\left\langle k \right\rangle=\left\{ 2,n^{\frac{1}{4}},n^{\frac{1}{2}} \right\}$^1^. The lower value $\left\langle k \right\rangle=2$ should produce a very sparse graph with primarily the largest-weighted edges but cannot be so sparse as to be likely strongly disconnected^1^. Afterwards, the directed graphs are replaced by three degree sequences (in-degree, out-degree and total degree) in both paths, while undirected graphs are replaced with their single degree sequences^1^. The aim of B&C’s simplifying process is to obtain simple networks for observation of scale-free structure.

Figure 1 Simplification paths of network data according to B&C

However, the dense weighted networks cannot be transformed into sparse unweighted networks by using LWE. To test this, we used the product network data from 2018. We followed the simplification process in B&C’s supplementary note and measured the number of nodes and mean degrees before and after simplification. Table 1 shows that the mean degrees after removing the largest-weighted edges (in the blue pane) are still much higher than the desired thresholds (in the orange pane). Obviously, LWE cannot generate sparse graphs. Furthermore, the number of nodes is drastically reduced compared with the number before simplification.

Table 1 Mean degrees and the number of nodes before and after simplification by LWE

| **Product code** | **Number of nodes before** | **Mean degree before** | **Desired mean degree (Thresholds of** $\mathbf{k}$**)** | **Number of links (Thresholds of** $\mathbf{m}$**)** | **Number of nodes after** | **Mean degree after** |
| --- | --- | --- | --- | --- | --- | --- |
| 0101 | 150 | 17.7067 | 2 | 150 | 47 | 6.3830 |
| 0101 | 150 | 17.7067 | 3 | 262 | 69 | 7.5362 |
| 0101 | 150 | 17.7067 | 12 | 919 | 120 | 14.4667 |
| 0102 | 145 | 11.3931 | 2 | 145 | 69 | 4.1739 |
| 0102 | 145 | 11.3931 | 3 | 252 | 84 | 6 |
| 0102 | 145 | 11.3931 | 12 | 873 | 145 | 11.3931 |
| 0103 | 110 | 8.1455 | 2 | 110 | 48 | 4.5833 |
| 0103 | 110 | 8.1455 | 3 | 178 | 63 | 5.5873 |
| 0103 | 110 | 8.1455 | 10 | 577 | 110 | 8.1455 |
| 0104 | 146 | 6.6301 | 2 | 146 | 69 | 4.1739 |
| 0104 | 146 | 6.6301 | 3 | 254 | 100 | 5.06 |
| 0104 | 146 | 6.6301 | 12 | 882 | 146 | 6.6301 |
| 0105 | 192 | 11.1146 | 2 | 192 | 91 | 4.2198 |
| 0105 | 192 | 11.1146 | 4 | 357 | 128 | 5.5781 |
| 0105 | 192 | 11.1146 | 14 | 1330 | 192 | 11.1146 |
| 0106 | 184 | 31.6522 | 2 | 184 | 65 | 5.6615 |
| 0106 | 184 | 31.6522 | 4 | 339 | 89 | 7.5730 |
| 0106 | 184 | 31.6522 | 14 | 1248 | 150 | 16.9867 |
| 0201 | 196 | 16.5306 | 2 | 196 | 66 | 5.9394 |
| 0201 | 196 | 16.5306 | 4 | 367 | 93 | 7.8925 |
| 0201 | 196 | 16.5306 | 14 | 1372 | 190 | 15.2105 |
| 0202 | 211 | 20.7678 | 2 | 211 | 81 | 5.2099 |
| 0202 | 211 | 20.7678 | 4 | 402 | 123 | 6.5366 |
| 0202 | 211 | 20.7678 | 15 | 1532 | 203 | 15.2709 |

Supplementary Note 2: Correlation between weights and network topology

We tested the correlation between strength and degree in TTN in 2018. In line with Barrat and Fagiolo^2,3^, we show the weight distribution, the strength distribution, the correlation between strength and degree, the behavior of the weighted clustering coefficient and the correlation between strength and the average nearest-neighbor strength for one product as an example in Figure 2-6.

1. The distribution of weights and the distribution of strengths follow an approximate power law.


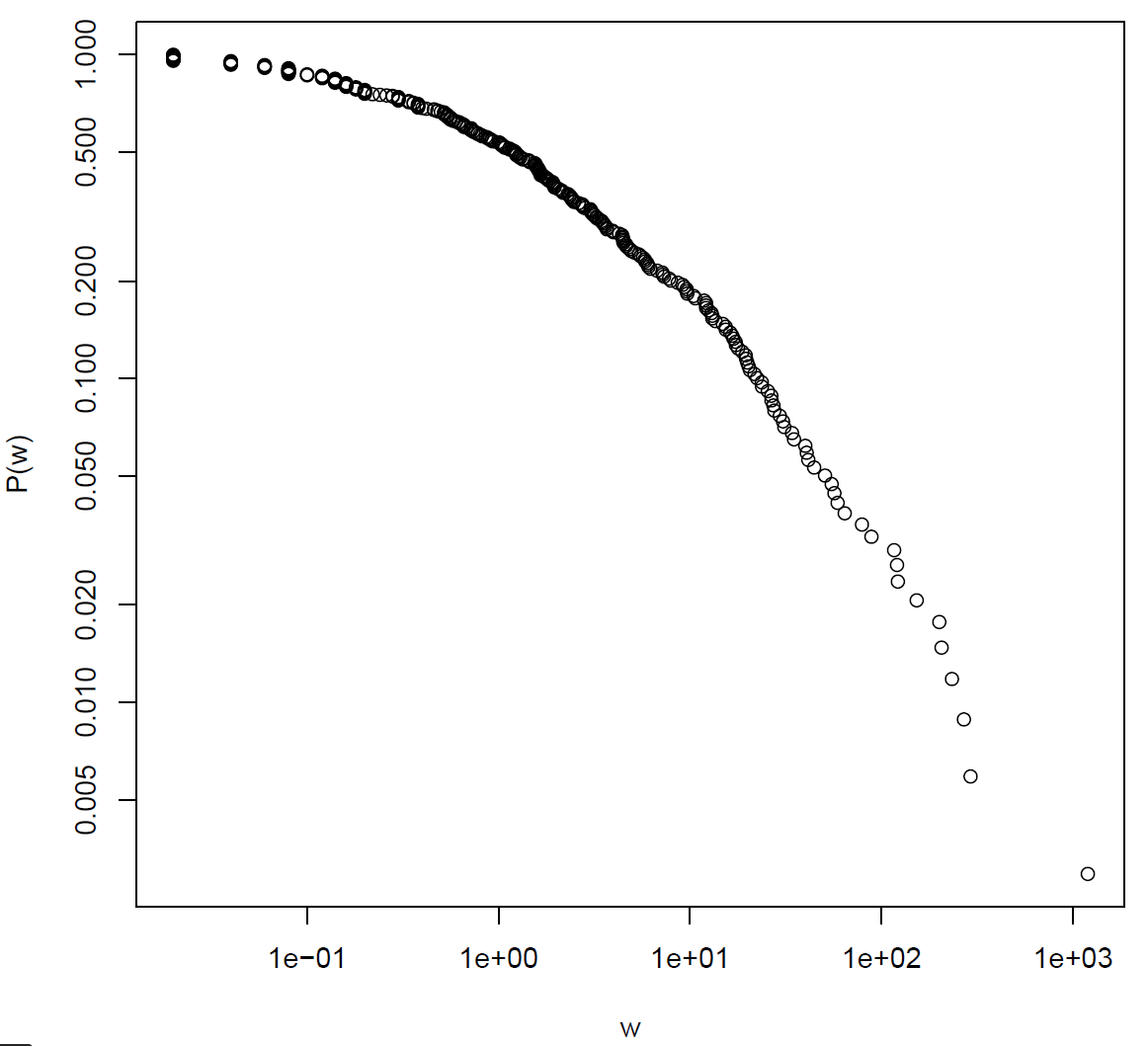


Figure 2 Distribution P(w) of weights (w) for product code 0101.


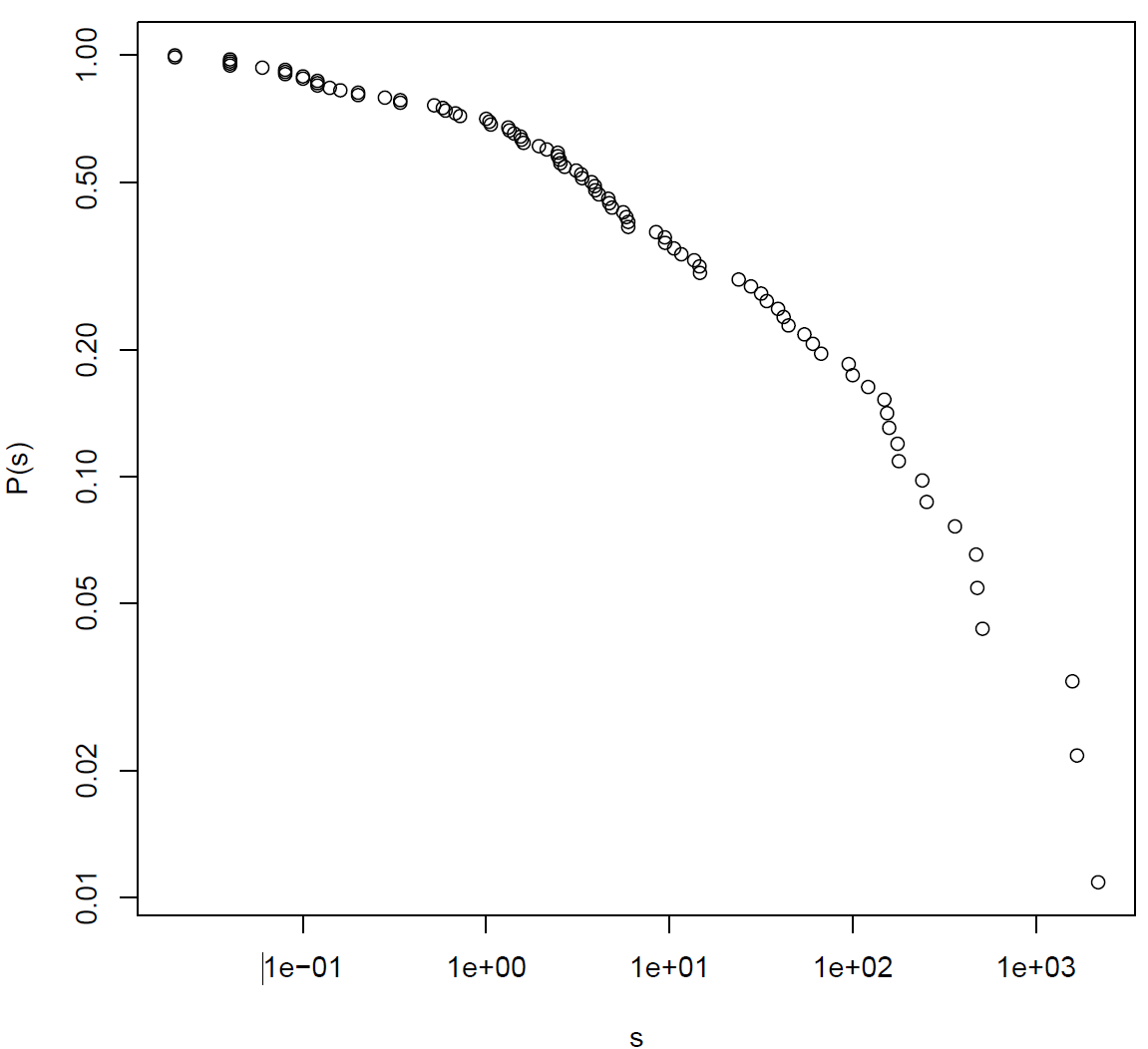


Figure 3 Distribution P(s) of strengths(s) for product code 0101.

1. Correlation: node strength grows faster than node degree.


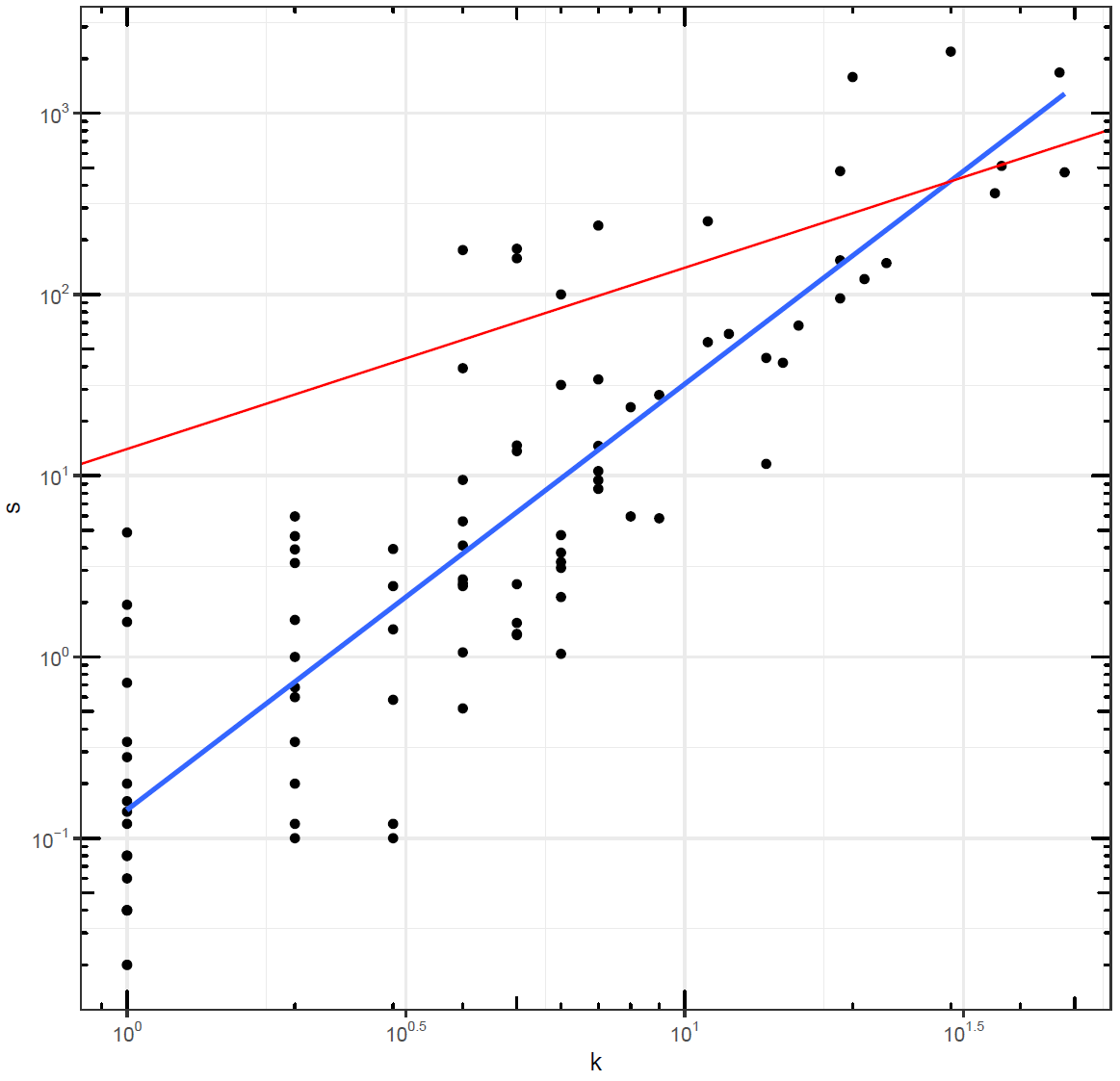


Figure 4 Strength(s) as a function of degree (k) for product code 0101. The blue line shows the results of linear regression fitting. The red line is defined for comparison and indicates that strength grows faster than degree.

1. Correlation: rich-club phenomenon.


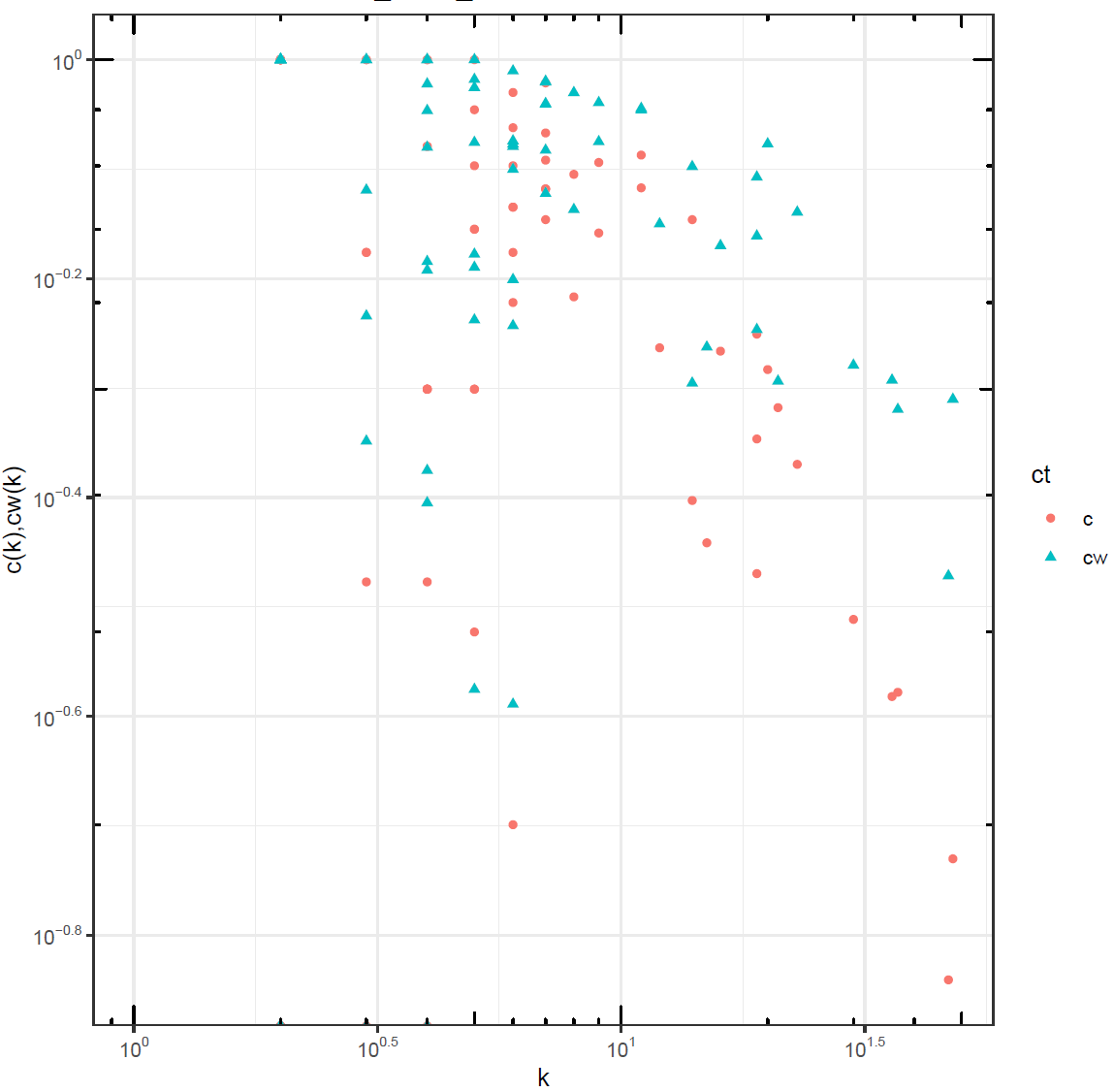


Figure 5 The weighted clustering coefficient cw(k) is larger than the topological one c(k) in the degree spectrum for product code 0101.


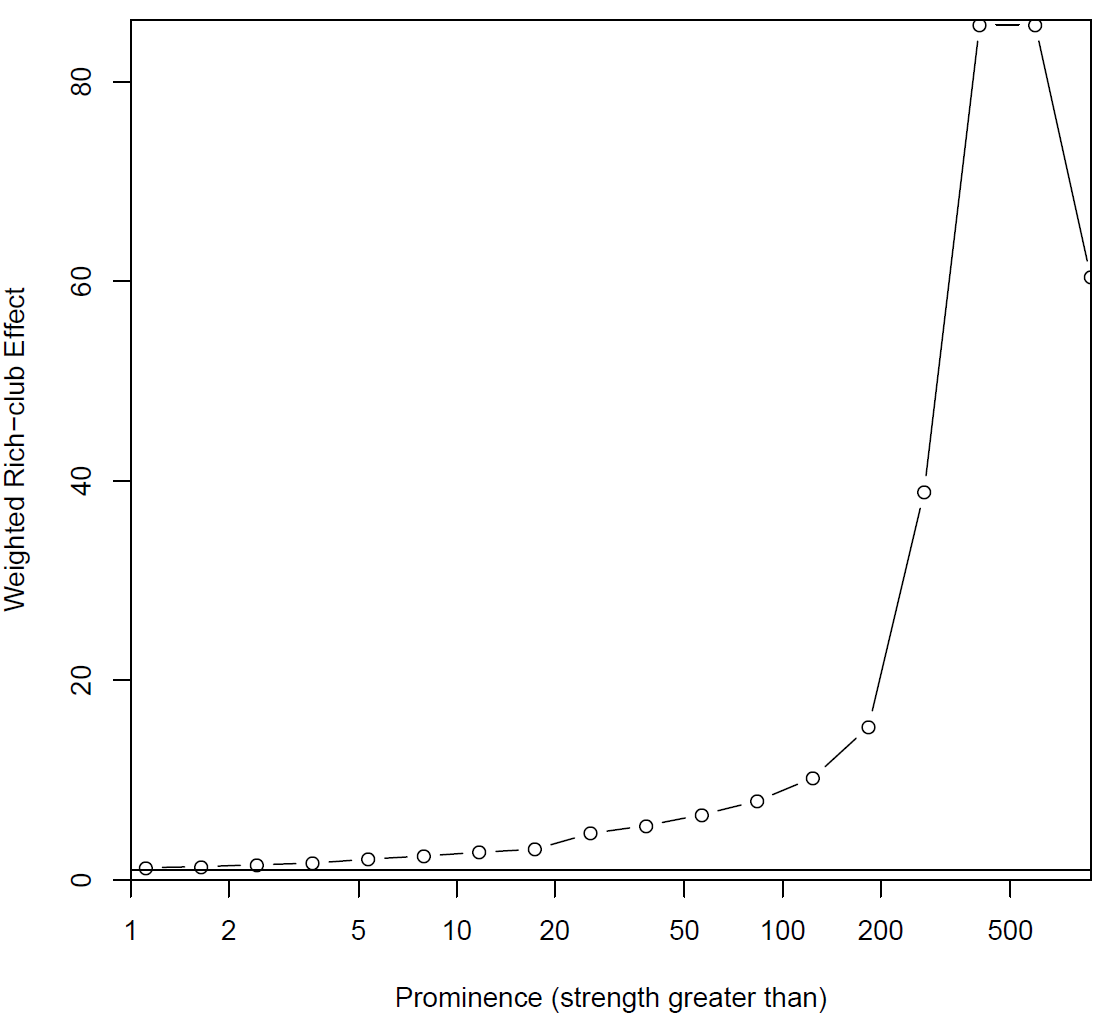


Figure 6 Prominence of weighted rich-club phenomenon in TTN for product code 0101.

Supplementary Note 3: Simplification by TN

There are 1225 simplified graphs for each of IMN, EXN and TTN in 2018. Similar to the last section, we measured the number of nodes and mean degrees before and after simplification. As shown in Table 2, Table 3 and Table 4, the resulting mean degrees are mostly two or close to two as desired. In addition, more nodes can be retained by using TN than by using LWE. Therefore, we argue that TN is superior for ITN to generate sparse graphs.

Table 2 Mean degrees and number of nodes before and after simplification by TN for IMN

| **Product code** | **Number of nodes before** | **Mean degree before** | **Number of nodes after** | **Mean degree after** |
| --- | --- | --- | --- | --- |
| 0101 | 150 | 17.7067 | 113 | 1.89 |
| 0102 | 145 | 11.3931 | 108 | 1.70 |
| 0103 | 110 | 8.1455 | 73 | 1.51 |
| 0104 | 146 | 6.6301 | 105 | 1.66 |
| 0105 | 192 | 11.1146 | 117 | 1.56 |
| 0106 | 184 | 31.6521 | 146 | 2 |
| 0201 | 196 | 16.5306 | 124 | 1.84 |
| 0202 | 211 | 20.7678 | 128 | 1.80 |

Table 3 Mean degrees and number of nodes before and after simplification by TN for EXN

| **Product code** | **Number of nodes before** | **Mean degree before** | **Number of nodes after** | **Mean degree after** |
| --- | --- | --- | --- | --- |
| 0101 | 150 | 17.7067 | 139 | 1.97 |
| 0102 | 145 | 11.3931 | 141 | 1.94 |
| 0103 | 110 | 8.1455 | 107 | 1.93 |
| 0104 | 146 | 6.6301 | 139 | 1.91 |
| 0105 | 192 | 11.1146 | 190 | 1.98 |
| 0106 | 184 | 31.6521 | 164 | 1.99 |
| 0201 | 196 | 16.5306 | 186 | 2 |
| 0202 | 211 | 20.7678 | 209 | 2 |

Table 4 Mean degrees and number of nodes before and after simplification by TN for TTN

| **Product code** | **Number of nodes before** | **Mean degree before** | **Number of nodes after** | **Mean degree after** |
| --- | --- | --- | --- | --- |
| 101 | 150 | 26.1333 | 150 | 2 |
| 102 | 145 | 18.6482 | 145 | 2 |
| 103 | 110 | 13.7091 | 110 | 2 |
| 104 | 146 | 11.3425 | 146 | 2 |
| 105 | 192 | 19.5625 | 192 | 2 |
| 106 | 184 | 49.0652 | 184 | 2 |
| 201 | 196 | 27.3265 | 196 | 2 |
| 202 | 211 | 35.2986 | 211 | 2 |

Supplementary Note 4: Definition of a scale-free network by B&C

Table 5 Definition of a scale-free network by B&C

|  | Fitting result of power law | Comparison to alternative distributions |
| --- | --- | --- |
| Super Weak | - | For at least 50% of graphs, no alternative distribution is favored over the power law. |
| Weakest | For at least 50% of graphs, a power-law distribution cannot be rejected (p ≥ 0.1). | - |
| Weak | On the basis of the weakest and the power-law region contains at least 50 nodes (n_tail_ ≥ 50). | - |
| Strong | On the basis of weak and 2 < $\hat{\alpha}$ < 3 for at least 50% of graphs. | For at least 50% of graphs, no alternative distribution is favored over the power law. |
| Strongest | Requirements of strong for at least 90% of graphs. | For at least 95% of graphs, no alternative distribution is favored over the power law. |

Supplementary Note 5: Results of the Entire Trade Network

Table 6 Test outcome for IMN as an entire trade network

| year | n_tail_ | α | p | LN_R | LN_p2 | EX_R | EX_p2 | PO_R | PO_p2 |
| --- | --- | --- | --- | --- | --- | --- | --- | --- | --- |
| 1995 | 212 | 2.590 | 0.009 | -2.175 | 0.030 | 3.332 | 0.001 | 2.421 | 0.015 |
| 1996 | 212 | 2.603 | 0.006 | -2.275 | 0.023 | 3.444 | 0.001 | 2.459 | 0.014 |
| 1997 | 212 | 2.574 | 0.006 | -2.409 | 0.016 | 3.349 | 0.001 | 2.395 | 0.017 |
| 1998 | 212 | 2.654 | 0.000 | -3.125 | 0.002 | 3.492 | 0.000 | 2.369 | 0.018 |
| 1999 | 38 | 2.083 | 0.713 | -3.125 | 0.002 | 3.492 | 0.000 | 2.369 | 0.018 |
| 2000 | 40 | 2.107 | 0.471 | -3.125 | 0.002 | 3.492 | 0.000 | 2.369 | 0.018 |
| 2001 | 215 | 2.580 | 0.075 | -1.375 | 0.169 | 2.782 | 0.005 | 1.999 | 0.046 |
| 2002 | 216 | 2.537 | 0.012 | -1.863 | 0.062 | 2.630 | 0.009 | 1.918 | 0.055 |
| 2003 | 216 | 2.564 | 0.088 | -1.330 | 0.184 | 2.609 | 0.009 | 1.899 | 0.058 |
| 2004 | 216 | 2.505 | 0.261 | -0.958 | 0.338 | 2.657 | 0.008 | 1.978 | 0.048 |
| 2005 | 217 | 2.496 | 0.472 | -0.634 | 0.526 | 2.695 | 0.007 | 2.050 | 0.040 |
| 2006 | 216 | 2.487 | 0.168 | -1.155 | 0.248 | 3.009 | 0.003 | 2.271 | 0.023 |
| 2007 | 216 | 2.488 | 0.318 | -0.901 | 0.368 | 2.809 | 0.005 | 2.138 | 0.033 |
| 2008 | 217 | 2.428 | 0.524 | -0.311 | 0.756 | 2.786 | 0.005 | 2.229 | 0.026 |
| 2009 | 217 | 2.495 | 0.008 | -2.001 | 0.045 | 3.323 | 0.001 | 2.496 | 0.013 |
| 2010 | 219 | 2.466 | 0.248 | -0.812 | 0.417 | 3.022 | 0.003 | 2.364 | 0.018 |
| 2011 | 221 | 2.478 | 0.114 | -1.354 | 0.176 | 3.280 | 0.001 | 2.548 | 0.011 |
| 2012 | 220 | 2.469 | 0.253 | -0.876 | 0.381 | 3.118 | 0.002 | 2.444 | 0.015 |
| 2013 | 220 | 2.422 | 0.696 | -0.117 | 0.907 | 2.746 | 0.006 | 2.317 | 0.021 |
| 2014 | 221 | 2.435 | 0.934 | -0.183 | 0.855 | 2.761 | 0.006 | 2.300 | 0.021 |
| 2015 | 221 | 2.503 | 0.134 | -1.269 | 0.204 | 3.054 | 0.002 | 2.345 | 0.019 |
| 2016 | 220 | 2.521 | 0.111 | -1.288 | 0.198 | 3.088 | 0.002 | 2.344 | 0.019 |
| 2017 | 49 | 2.188 | 0.487 | -1.288 | 0.198 | 3.088 | 0.002 | 2.344 | 0.019 |
| 2018 | 221 | 2.514 | 0.085 | -1.369 | 0.171 | 3.262 | 0.001 | 2.513 | 0.012 |

Table 7 Test outcome for EXN as an entire trade network

| year | n_tail_ | α | p | LN_R | LN_p2 | EX_R | EX_p2 | PO_R | PO_p2 |
| --- | --- | --- | --- | --- | --- | --- | --- | --- | --- |
| 1995 | 212 | 2.635 | 0.015 | -2.044 | 0.041 | 3.841 | 0.000 | 2.756 | 0.006 |
| 1996 | 213 | 2.650 | 0.000 | -3.218 | 0.001 | 4.093 | 0.000 | 2.817 | 0.005 |
| 1997 | 212 | 2.658 | 0.000 | -3.330 | 0.001 | 4.063 | 0.000 | 2.786 | 0.005 |
| 1998 | 213 | 2.627 | 0.000 | -2.854 | 0.004 | 3.787 | 0.000 | 2.655 | 0.008 |
| 1999 | 36 | 1.984 | 0.816 | -2.854 | 0.004 | 3.787 | 0.000 | 2.655 | 0.008 |
| 2000 | 216 | 2.577 | 0.003 | -2.426 | 0.015 | 3.730 | 0.000 | 2.637 | 0.008 |
| 2001 | 216 | 2.588 | 0.001 | -2.638 | 0.008 | 3.582 | 0.000 | 2.488 | 0.013 |
| 2002 | 39 | 2.036 | 0.416 | -2.638 | 0.008 | 3.582 | 0.000 | 2.488 | 0.013 |
| 2003 | 216 | 2.571 | 0.022 | -1.868 | 0.062 | 3.568 | 0.000 | 2.601 | 0.009 |
| 2004 | 216 | 2.557 | 0.013 | -2.024 | 0.043 | 3.733 | 0.000 | 2.740 | 0.006 |
| 2005 | 217 | 2.571 | 0.000 | -3.608 | 0.000 | 4.206 | 0.000 | 2.969 | 0.003 |
| 2006 | 217 | 2.512 | 0.026 | -1.748 | 0.081 | 3.506 | 0.000 | 2.651 | 0.008 |
| 2007 | 217 | 2.551 | 0.006 | -2.274 | 0.023 | 3.962 | 0.000 | 2.895 | 0.004 |
| 2008 | 217 | 2.521 | 0.013 | -1.875 | 0.061 | 3.517 | 0.000 | 2.680 | 0.007 |
| 2009 | 217 | 2.542 | 0.004 | -2.273 | 0.023 | 3.556 | 0.000 | 2.646 | 0.008 |
| 2010 | 219 | 2.547 | 0.005 | -2.430 | 0.015 | 3.458 | 0.001 | 2.534 | 0.011 |
| 2011 | 221 | 2.563 | 0.000 | -3.438 | 0.001 | 4.053 | 0.000 | 2.868 | 0.004 |
| 2012 | 220 | 2.551 | 0.004 | -2.493 | 0.013 | 3.384 | 0.001 | 2.449 | 0.014 |
| 2013 | 221 | 2.542 | 0.006 | -2.299 | 0.022 | 3.420 | 0.001 | 2.513 | 0.012 |
| 2014 | 221 | 2.546 | 0.023 | -1.834 | 0.067 | 2.935 | 0.003 | 2.184 | 0.029 |
| 2015 | 221 | 2.651 | 0.002 | -2.357 | 0.018 | 2.808 | 0.005 | 1.989 | 0.047 |
| 2016 | 221 | 2.660 | 0.000 | -2.859 | 0.004 | 2.943 | 0.003 | 2.075 | 0.038 |
| 2017 | 221 | 2.629 | 0.010 | -1.944 | 0.052 | 2.849 | 0.004 | 2.071 | 0.038 |
| 2018 | 221 | 2.665 | 0.000 | -2.710 | 0.007 | 2.853 | 0.004 | 2.023 | 0.043 |

Table 8 Test outcome for TTN as an entire trade network

| year | n_tail_ | α | p | LN_R | LN_p2 | EX_R | EX_p2 | PO_R | PO_p2 |
| --- | --- | --- | --- | --- | --- | --- | --- | --- | --- |
| 1995 | 212 | 2.642 | 0.020 | -1.900 | 0.057 | 3.348 | 0.001 | 2.390 | 0.017 |
| 1996 | 213 | 2.689 | 0.000 | -3.294 | 0.001 | 3.615 | 0.000 | 2.432 | 0.015 |
| 1997 | 213 | 2.645 | 0.000 | -2.956 | 0.003 | 3.559 | 0.000 | 2.427 | 0.015 |
| 1998 | 213 | 2.704 | 0.000 | -3.150 | 0.002 | 3.327 | 0.001 | 2.240 | 0.025 |
| 1999 | 210 | 2.689 | 0.000 | -3.925 | 0.000 | 3.452 | 0.001 | 2.305 | 0.021 |
| 2000 | 216 | 2.640 | 0.000 | -3.199 | 0.001 | 3.293 | 0.001 | 2.229 | 0.026 |
| 2001 | 216 | 2.668 | 0.000 | -3.610 | 0.000 | 3.326 | 0.001 | 2.214 | 0.027 |
| 2002 | 216 | 2.659 | 0.000 | -3.663 | 0.000 | 3.574 | 0.000 | 2.390 | 0.017 |
| 2003 | 216 | 2.658 | 0.000 | -2.962 | 0.003 | 3.385 | 0.001 | 2.298 | 0.022 |
| 2004 | 216 | 2.602 | 0.000 | -3.070 | 0.002 | 3.519 | 0.000 | 2.428 | 0.015 |
| 2005 | 217 | 2.627 | 0.000 | -3.971 | 0.000 | 3.812 | 0.000 | 2.550 | 0.011 |
| 2006 | 217 | 2.571 | 0.001 | -2.602 | 0.009 | 3.512 | 0.000 | 2.478 | 0.013 |
| 2007 | 217 | 2.627 | 0.000 | -3.834 | 0.000 | 3.674 | 0.000 | 2.529 | 0.011 |
| 2008 | 217 | 2.562 | 0.013 | -2.033 | 0.042 | 3.376 | 0.001 | 2.475 | 0.013 |
| 2009 | 40 | 2.037 | 0.491 | -2.033 | 0.042 | 3.376 | 0.001 | 2.475 | 0.013 |
| 2010 | 219 | 2.590 | 0.001 | -3.411 | 0.001 | 3.648 | 0.000 | 2.605 | 0.009 |
| 2011 | 221 | 2.603 | 0.000 | -3.419 | 0.001 | 3.771 | 0.000 | 2.654 | 0.008 |
| 2012 | 220 | 2.634 | 0.000 | -4.400 | 0.000 | 3.723 | 0.000 | 2.560 | 0.010 |
| 2013 | 221 | 2.608 | 0.000 | -3.609 | 0.000 | 3.314 | 0.001 | 2.328 | 0.020 |
| 2014 | 221 | 2.598 | 0.000 | -3.013 | 0.003 | 3.165 | 0.002 | 2.247 | 0.025 |
| 2015 | 221 | 2.709 | 0.000 | -3.166 | 0.002 | 3.027 | 0.002 | 2.106 | 0.035 |
| 2016 | 221 | 2.701 | 0.000 | -3.633 | 0.000 | 3.164 | 0.002 | 2.186 | 0.029 |
| 2017 | 221 | 2.674 | 0.000 | -3.316 | 0.001 | 3.115 | 0.002 | 2.175 | 0.030 |
| 2018 | 222 | 2.695 | 0.000 | -3.068 | 0.002 | 2.919 | 0.004 | 2.029 | 0.042 |

Supplementary Note 6: Results of Alternative Distributions

Table 9 Test outcomes of the comparison of scale-free and alternative distributions for EXN. The percentage of network data sets that favor the power-law model M_PL_, alternative model M_LN_, M_EX_, M_PO_ or neither as M_inconclusive_ under a likelihood-ratio test are shown.

|  | compared to log-normal distribution | | | compared to exponential distribution | | | compared to Poisson distribution | | | |
| --- | --- | --- | --- | --- | --- | --- | --- | --- | --- | --- |
| year | M_PL_ | M_LN_ | M_inconclusive_ | M_PL_ | M_EX_ | M_inconclusive_ | M_PL_ | M_PO_ | M_inconclusive_ |  |
| 1995 | 0% | 54% | 46% | 91% | 0% | 9% | 88% | 0% | 12% |  |
| 1996 | 0% | 56% | 44% | 91% | 0% | 9% | 88% | 0% | 12% |  |
| 1997 | 0% | 52% | 48% | 91% | 0% | 9% | 88% | 0% | 12% |  |
| 1998 | 0% | 54% | 46% | 91% | 0% | 9% | 88% | 0% | 12% |  |
| 1999 | 0% | 55% | 45% | 91% | 0% | 9% | 88% | 0% | 12% |  |
| 2000 | 0% | 52% | 48% | 91% | 0% | 9% | 87% | 0% | 13% |  |
| 2001 | 0% | 50% | 50% | 90% | 0% | 9% | 86% | 0% | 14% |  |
| 2002 | 0% | 49% | 51% | 89% | 0% | 11% | 84% | 0% | 16% |  |
| 2003 | 0% | 49% | 51% | 90% | 0% | 10% | 85% | 0% | 15% |  |
| 2004 | 0% | 47% | 53% | 90% | 0% | 10% | 84% | 0% | 16% |  |
| 2005 | 0% | 49% | 51% | 90% | 0% | 10% | 84% | 0% | 16% |  |
| 2006 | 0% | 47% | 53% | 89% | 0% | 11% | 80% | 0% | 20% |  |
| 2007 | 0% | 48% | 52% | 90% | 0% | 10% | 81% | 0% | 19% |  |
| 2008 | 0% | 46% | 54% | 90% | 0% | 10% | 79% | 0% | 21% |  |
| 2009 | 0% | 44% | 56% | 89% | 0% | 11% | 77% | 0% | 23% |  |
| 2010 | 0% | 42% | 58% | 89% | 0% | 11% | 75% | 0% | 25% |  |
| 2011 | 0% | 44% | 56% | 89% | 0% | 11% | 75% | 0% | 25% |  |
| 2012 | 0% | 49% | 51% | 90% | 0% | 10% | 74% | 0% | 26% |  |
| 2013 | 0% | 46% | 54% | 89% | 0% | 11% | 73% | 0% | 27% |  |
| 2014 | 0% | 42% | 58% | 88% | 0% | 12% | 70% | 0% | 30% |  |
| 2015 | 0% | 47% | 53% | 89% | 0% | 11% | 69% | 0% | 31% |  |
| 2016 | 0% | 48% | 52% | 88% | 0% | 12% | 69% | 0% | 31% |  |
| 2017 | 0% | 48% | 52% | 89% | 0% | 11% | 70% | 0% | 30% |  |
| 2018 | 0% | 53% | 47% | 89% | 0% | 11% | 69% | 0% | 31% |  |

Table 10 Test outcomes of the comparison of scale-free and alternative distributions for IMN. The percentage of network data sets that favor the power-law model M_PL_, alternative model M_LN_, M_EX_, M_PO_ or neither as M_inconclusive_ under a likelihood-ratio test are shown.

|  | compared to log-normal distribution | | | compared to exponential distribution | | | compared to Poisson distribution | | | |
| --- | --- | --- | --- | --- | --- | --- | --- | --- | --- | --- |
| year | M_PL_ | M_LN_ | M_inconclusive_ | M_PL_ | M_EX_ | M_inconclusive_ | M_PL_ | M_PO_ | M_inconclusive_ |  |
| 1995 | 0% | 54% | 46% | 91% | 0% | 9% | 88% | 0% | 12% |  |
| 1996 | 0% | 56% | 44% | 91% | 0% | 9% | 88% | 0% | 12% |  |
| 1997 | 0% | 52% | 48% | 91% | 0% | 9% | 88% | 0% | 12% |  |
| 1998 | 0% | 54% | 46% | 91% | 0% | 9% | 88% | 0% | 12% |  |
| 1999 | 0% | 55% | 45% | 91% | 0% | 9% | 88% | 0% | 12% |  |
| 2000 | 0% | 52% | 48% | 91% | 0% | 9% | 87% | 0% | 13% |  |
| 2001 | 0% | 50% | 50% | 90% | 0% | 9% | 86% | 0% | 14% |  |
| 2002 | 0% | 49% | 51% | 89% | 0% | 11% | 84% | 0% | 16% |  |
| 2003 | 0% | 49% | 51% | 90% | 0% | 10% | 85% | 0% | 15% |  |
| 2004 | 0% | 47% | 53% | 90% | 0% | 10% | 84% | 0% | 16% |  |
| 2005 | 0% | 49% | 51% | 90% | 0% | 10% | 84% | 0% | 16% |  |
| 2006 | 0% | 47% | 53% | 89% | 0% | 11% | 80% | 0% | 20% |  |
| 2007 | 0% | 48% | 52% | 90% | 0% | 10% | 81% | 0% | 19% |  |
| 2008 | 0% | 46% | 54% | 90% | 0% | 10% | 79% | 0% | 21% |  |
| 2009 | 0% | 44% | 56% | 89% | 0% | 11% | 77% | 0% | 23% |  |
| 2010 | 0% | 42% | 58% | 89% | 0% | 11% | 75% | 0% | 25% |  |
| 2011 | 0% | 44% | 56% | 89% | 0% | 11% | 75% | 0% | 25% |  |
| 2012 | 0% | 49% | 51% | 90% | 0% | 10% | 74% | 0% | 26% |  |
| 2013 | 0% | 46% | 54% | 89% | 0% | 11% | 73% | 0% | 27% |  |
| 2014 | 0% | 42% | 58% | 88% | 0% | 12% | 70% | 0% | 30% |  |
| 2015 | 0% | 47% | 53% | 89% | 0% | 11% | 69% | 0% | 31% |  |
| 2016 | 0% | 48% | 52% | 88% | 0% | 12% | 69% | 0% | 31% |  |
| 2017 | 0% | 48% | 52% | 89% | 0% | 11% | 70% | 0% | 30% |  |
| 2018 | 0% | 53% | 47% | 89% | 0% | 11% | 69% | 0% | 31% |  |

Table 11 Test outcomes of the comparison of scale-free and alternative distributions for TTN. The percentage of network data sets that favor the power-law model M_PL_, alternative model M_LN_, M_EX_, M_PO_ or neither as M_inconnlusive_ under a likelihood-ratio test are shown.

|  | compared to log-normal distribution | | | compared to exponential distribution | | | compared to Poisson distribution | | | |
| --- | --- | --- | --- | --- | --- | --- | --- | --- | --- | --- |
| year | M_PL_ | M_LN_ | M_inconclusive_ | M_PL_ | M_EX_ | M_inconclusive_ | M_PL_ | M_PO_ | M_inconclusive_ |  |
| 1995 | 0% | 28% | 72% | 88% | 0% | 12% | 91% | 0% | 9% |  |
| 1996 | 0% | 28% | 72% | 86% | 0% | 14% | 90% | 0% | 10% |  |
| 1997 | 0% | 24% | 76% | 90% | 0% | 10% | 95% | 0% | 5% |  |
| 1998 | 0% | 22% | 78% | 87% | 0% | 13% | 89% | 0% | 11% |  |
| 1999 | 0% | 23% | 77% | 87% | 0% | 13% | 89% | 0% | 11% |  |
| 2000 | 0% | 21% | 79% | 88% | 0% | 12% | 94% | 0% | 6% |  |
| 2001 | 0% | 22% | 78% | 84% | 0% | 16% | 86% | 0% | 14% |  |
| 2002 | 0% | 19% | 81% | 84% | 0% | 16% | 85% | 0% | 15% |  |
| 2003 | 0% | 19% | 81% | 86% | 0% | 14% | 87% | 0% | 13% |  |
| 2004 | 0% | 17% | 83% | 90% | 0% | 10% | 93% | 0% | 7% |  |
| 2005 | 0% | 17% | 83% | 88% | 0% | 12% | 93% | 0% | 7% |  |
| 2006 | 0% | 15% | 85% | 84% | 0% | 16% | 84% | 0% | 16% |  |
| 2007 | 0% | 14% | 86% | 84% | 0% | 16% | 83% | 0% | 17% |  |
| 2008 | 0% | 14% | 86% | 89% | 0% | 11% | 91% | 0% | 9% |  |
| 2009 | 0% | 11% | 89% | 88% | 0% | 12% | 89% | 0% | 11% |  |
| 2010 | 0% | 11% | 89% | 88% | 0% | 12% | 86% | 0% | 14% |  |
| 2011 | 0% | 11% | 89% | 87% | 0% | 13% | 86% | 0% | 14% |  |
| 2012 | 0% | 14% | 86% | 87% | 0% | 13% | 86% | 0% | 14% |  |
| 2013 | 0% | 12% | 88% | 82% | 0% | 18% | 78% | 0% | 22% |  |
| 2014 | 0% | 14% | 85% | 87% | 0% | 12% | 84% | 0% | 15% |  |
| 2015 | 0% | 14% | 85% | 84% | 0% | 15% | 78% | 0% | 22% |  |
| 2016 | 0% | 16% | 84% | 84% | 0% | 16% | 78% | 0% | 22% |  |
| 2017 | 0% | 15% | 85% | 83% | 0% | 17% | 77% | 0% | 23% |  |
| 2018 | 0% | 19% | 81% | 87% | 0% | 13% | 80% | 0% | 20% |  |

**References**

1. Broido, A. D. & Clauset, A. Supplementary Information: Scale-free networks are rare. *Nature Communications* **10**, 1017 (2019).

2. Barrat, A., Barthelemy, M., Pastor-Satorras, R. & Vespignani, A. The architecture of complex weighted networks. *Proceedings of the National Academy of Sciences* **101**, 3747–3752 (2004).

3. Fagiolo, G., Reyes, J. & Schiavo, S. On the topological properties of the world trade web: A weighted network analysis. *Physica A: Statistical Mechanics and its Applications* **387**, 3868–3873 (2008).
